# A Circulating Proteomic Signature of Allostatic Load Predicts Multisystem Disease and Mortality

**DOI:** 10.21203/rs.3.rs-8881432/v1

**Published:** 2026-04-16

**Authors:** Yufan Guan, Jie Shen, Li Li, Kai Zhang, Song Liu, Qianqian Zhu, Philip I-Fon Chow, Hongmei Jiang, Jing Li, Lifang Hou, Hua Zhao

**Affiliations:** 1. Department of Public Health Sciences, School of Medicine, University of Virginia, Charlottesville, VA 22903, United States; 2. Department of Family Medicine, School of Medicine, University of Virginia, Charlottesville, VA 22903, United States; 3. Departments of Population and Community Health, College of Public Health, University of North Texas Health Science Center, Fort Worth, TX 76107, United States; 4. Department of Biostatistics and Bioinformatics, Roswell Park Cancer Institute, Buffalo, NY, 14263, United States; 5. Department of Psychiatry and Neurobehavioral Sciences, School of Medicine, University of Virginia, Charlottesville, VA 22903, United States; 6. Department of Statistics and Data Science, Northwestern University, Evanston, IL, 60208, United States; 7. Division of General Internal Medicine & Population Science, University of Alabama at Birmingham, Birmingham, AL, 35233, United States; 8. Department of Preventive Medicine, Feinberg School of Medicine, Northwestern University, Chicago, IL, 60611, United States

## Abstract

Chronic stress contributes to the development of cardiometabolic, malignant, and other chronic diseases through cumulative multisystem physiological dysregulation, conceptualized as allostatic load (AL). However, traditional AL relies on heterogeneous clinical biomarkers that limit reproducibility and translational utility. Here, we develop and validate ProAL50, a proteomics-based measure of AL derived from 50 circulating proteins. Using high-dimensional plasma proteomic data from the UK Biobank, we constructed ProAL50 via penalized regression and stability selection and externally validated it in the Coronary Artery Risk Development in Young Adults (CARDIA) Study. ProAL50 closely mirrored traditional AL in its associations with sociodemographic characteristics, lifestyle behaviors, physical and mental health, inflammation, and biological aging, supporting strong construct validity. Beyond replication, ProAL50 consistently demonstrated stronger associations with incident chronic diseases, including all cancers, type 2 diabetes, ischemic heart disease, chronic lung disease, and chronic kidney disease, and with all-cause and cause-specific mortality. Functional enrichment analyses revealed that ProAL50 proteins cluster within lipid metabolic and immune–inflammatory pathways. These findings establish ProAL50 as a scalable, biologically grounded measure of cumulative stress that not only replaces traditional AL but surpasses it in predicting disease risk and mortality, offering a novel tool for population health and translational research.

## Introduction

Chronic stress is increasingly recognized as a fundamental determinant of population health, influencing the onset and progression of cardiovascular, metabolic, neuropsychiatric, and malignant diseases. The biological imprint of chronic stress is conceptualized through allostasis and allostatic load (AL), a multisystem index of cumulative physiological dysregulation across neuroendocrine, metabolic, inflammatory, and cardiovascular pathways (1, 2). AL has been widely used in epidemiologic studies and consistently predicts frailty, multimorbidity, functional decline, incident chronic disease, and mortality (3–6).

Despite its conceptual significance, the practical application of traditional AL has been constrained by several methodological and translational limitations. Traditional AL indices rely on clinical laboratory biomarkers such as blood pressure, lipid profiles, glucose metabolism, inflammatory markers, and anthropometric measures. These biomarkers are often collected inconsistently across studies, require fasting, vary across laboratories, and frequently exhibit missingness in real-world datasets. Moreover, there is no standardized algorithm for selecting biomarkers or defining high-risk cut-points; studies differ widely in biomarker inclusion, scoring method, and weighting, undermining reproducibility and comparability (7–9). These limitations impede clinical translation and highlight the need for more biologically grounded and standardized measures.

Advances in high-throughput proteomics offer an opportunity to modernize the measurement of AL. Circulating proteins integrate genetic, environmental, behavioral, and contextual influences and capture dynamic, system-wide physiological states. Large-scale proteomic platforms such as Olink Explore panels provide standardized, reproducible quantification of thousands of plasma proteins with minimal sample requirements. Emerging work demonstrates that proteomic signatures can robustly index biological aging, systemic inflammation, immune activation, and chronic disease risk (10–13). For example, proteomics-based aging clocks outperform many clinical biomarkers in predicting morbidity and mortality (10, 12). These features make proteomics particularly well suited for capturing the multisystem dysregulation that underlies chronic stress.

Building on advances in high-throughput proteomics, we developed ProAL50, a proteomics-based measure of AL derived from circulating plasma proteins in a large sample from the UK Biobank (UKB; *n* = 53,013) and externally validated the model in the Coronary Artery Risk Development in Young Adults (CARDIA) study using Year 25 (Y25) data (*n* = 2,796). We then systematically evaluated associations between ProAL50 and sociodemographic factors, lifestyle behaviors, physical and mental health, biological aging, incident chronic diseases, site-specific cancers, and cause-specific mortality. Finally, we characterized the biological pathways captured by ProAL50 using functional enrichment and protein–protein interaction analyses.

## Results

### ProAL50: a proteomic signature of AL

A schematic representation of the study design and main analytic approaches is shown in Fig. 1. The UK Biobank cohort was randomly split into a 70% training set and a 30% internal test set for proteomic score development and validation (Fig. 1a). In the training phase, LASSO/elastic net models were fitted to predict traditional AL, and protein features were retained based on their stability across 1,000 bootstrap resamples. Internal validation of ProAL50 was performed within the UK Biobank by comparing the proteomic score with multiple established AL constructs in the independent test set (Fig 1b). External validation was performed using an independent cohort from the CARDIA Study (Year 25). The downstream application of ProAL50 is illustrated in Fig. 1c, which outlines the analytic framework used to relate ProAL50 to demographic characteristics, socioeconomic status, lifestyle factors, general and mental health measures, chronic disease burden, cancer incidence, and mortality outcomes.

Using high-dimensional plasma proteomic data from the Olink Explore platform (2,922 proteins), we developed ProAL50 through penalized regression and bootstrap stability selection, yielding a consensus panel of the top 50 proteins. Model performance in the training data was evaluated by correlating predicted and observed AL, demonstrating strong predictive accuracy (Pearson *r* = 0.72, p < 0.001, Fig. 2a). Internal validation further supported the robustness of ProAL50, showing high concordance with quartile-based AL (Pearson *r* = 0.73, p < 0.001; Fig. 2b) and with a reduced AL panel (Pearson *r* = 0.70, p < 0.001; Fig. 2c). External validation in an independent cohort from the CARDIA Study (Year 25) demonstrated strong generalizability (Pearson r = 0.62, p < 0.001; Fig. 2d).

### Sociodemographic and lifestyle correlates of ProAL50

In analyses of sociodemographic and lifestyle determinants (Table 1), ProAL50 demonstrated patterns of association highly consistent with those observed for traditional AL. Older age (>57 years) showed the strongest demographic association with both measures, followed by male sex. Racial differences were modest: compared with White participants, Asian participants exhibited higher AL and ProAL50, whereas Black participants showed no difference in AL but lower ProAL50. Indicators of higher socioeconomic status, including higher household income and educational attainment, were consistently associated with lower AL and ProAL50, while greater neighborhood deprivation (higher Townsend index) was associated with higher values of both measures.

Unhealthy lifestyle behaviors were uniformly associated with higher stress-related burden. Both former and current smoking were associated with higher AL and ProAL50 compared with never smoking, whereas alcohol consumption showed a graded inverse association, with moderate and heavy drinkers exhibiting lower values than never drinkers. Physical activity showed the strongest protective association, with moderate and high activity levels associated with substantially lower AL and ProAL50 in a dose–response manner. Sleep disturbance was positively associated with both indices, with progressively higher values observed among participants reporting occasional or frequent sleeplessness.

### General and Mental Health Associated with ProAL50

Measures of general and mental health showed strong, graded, and largely concordant associations with AL and ProAL50 (Fig. 3 and **Supplementary Table 2**). Better physical functioning and self-rated health were consistently associated with lower AL across indices. Compared with participants reporting a slow walking pace, those reporting a brisk pace exhibited substantially lower AL (β = −0.90, 95% CI −0.97 to −0.84) and ProAL50 (β = −0.16, 95% CI −0.17 to −0.15). Similarly, excellent self-rated health was strongly associated with lower stress-related burden compared with poor health (AL: β = −1.20, 95% CI −1.28 to −1.11; ProAL50: β = −0.20, 95% CI −0.22 to −0.19).

In contrast, markers of poorer health showed parallel positive associations. Greater fatigue frequency was associated with higher AL and ProAL50, with participants reporting near-daily fatigue exhibiting higher AL (β = 0.38, 95% CI 0.31 to 0.45) and ProAL50 (β = 0.07, 95% CI 0.06 to 0.09) compared with those reporting no fatigue. Individuals with a positive ProAgeGap (≥0) also had higher AL (β = 0.14, 95% CI 0.10 to 0.17) and ProAL50 (β = 0.04, 95% CI 0.03 to 0.05). Inflammatory and mental health markers showed similar patterns: elevated NLR (≥3) was associated with higher AL (β = 0.12, 95% CI 0.08 to 0.16), although the corresponding association with ProAL50 was weaker and not statistically significant. Participants with anxiety had higher AL and ProAL50, with comparable associations observed for depression and comorbid anxiety/depression.

### ProAL50 Predicts Risk of Common Chronic Diseases

Higher AL and ProAL50 were consistently associated with increased chronic disease risk, with the strongest and most robust associations observed for all cancers, type-2 diabetes, chronic lung disease, and chronic kidney disease (Fig. 4 and **Supplementary Table 3**). For overall cancer, both measures showed significant associations that remained after full adjustment (AL HR = 1.04, 95% CI 1.02–1.06; ProAL50 HR = 1.06, 95% CI 1.03–1.10). In site-specific analyses, associations varied by cancer type (**Supplementary Table 4**). Increased AL and ProAL50 were significantly associated with elevated risks of bowel, kidney, and cervical cancers. In addition, higher ProAL50 was associated with an increased risk of liver cancer.

For type 2 diabetes (N = 3,458), both AL and ProAL50 demonstrated strong and highly significant adjusted associations (AL: HR = 1.06, 95% CI 1.04–1.09; ProAL50: HR = 1.08, 95% CI 1.04–1.12). Associations with ischemic heart disease (N = 4,873) were largely attenuated for AL after adjustment, whereas ProAL50 remained modestly predictive (HR = 1.05, 95% CI 1.01–1.09). No adjusted associations were observed for cerebrovascular disease (N = 2,090). For emphysema/COPD (N = 2,059), both measures remained significantly associated after adjustment (AL: HR = 1.04, 95% CI 1.01–1.07; ProAL50: HR = 1.08, 95% CI 1.04–1.12), while neither predicted chronic liver disease incidence (N = 833). Strong concordant associations were observed for chronic kidney disease (N = 2,170), with both AL (HR = 1.07, 95% CI 1.04–1.10) and ProAL50 (HR = 1.08, 95% CI 1.04–1.12) remaining significant predictors. In contrast, neither biomarker showed significant adjusted associations with Alzheimer’s disease, Parkinson’s disease, or parkinsonism.

### ProAL50 Predicts All-Cause and Cause-Specific Mortality

Higher AL and ProAL50 were consistently associated with increased risk of all-cause and cause-specific mortality, with generally stronger associations observed for ProAL50 (Fig. 5 and **Supplementary Table 5**). For all-cause mortality (N = 3,650), both measures showed robust associations in crude and fully adjusted models, with adjusted hazard ratios of 1.11 (95% CI 1.08–1.13) for AL and 1.25 (95% CI 1.20–1.30) for ProAL50. Similar patterns were observed for overall cancer mortality (N = 1,448), with significant associations for both AL (HR = 1.08, 95% CI 1.04–1.11) and ProAL50 (HR = 1.23, 95% CI 1.16–1.31).

In site-specific cancer mortality analyses, associations varied by cancer type and were stronger for ProAL50 (**Supplementary Table 6**). After adjusting for covariates and multiple comparisons, ProAL50 was significantly associated with mortality from breast, bowel, liver, and non-Hodgkin lymphoma, while the association for kidney cancer mortality was marginally significant. On the other hand, no statistically significant association was observed for AL.

For non-cancer chronic diseases, both biomarkers demonstrated strong and concordant associations with metabolic and vascular mortality. Type 2 diabetes mortality (N = 16) showed particularly large effect sizes for both AL (HR = 2.67, 95% CI 1.86–3.81) and ProAL50 (HR per SD = 3.89, 95% CI 2.16–7.00). Cerebrovascular disease mortality (N = 79) and ischemic heart disease mortality (N = 403) were also significantly associated with both measures after adjustment, with consistently stronger associations for ProAL50. In contrast, associations with emphysema/COPD mortality were attenuated and no longer statistically significant after adjustment.

### Biological functions and protein interaction networks

To characterize the biological functions represented by the 50-protein panel, we performed functional enrichment analysis using the STRING database. The protein list was mapped to human genes/proteins in STRING, and overrepresentation analysis was conducted for Gene Ontology (GO) biological process terms. Enrichment significance was evaluated using FDR correction, and the top significantly enriched terms were reported (**Supplementary Table 7–12**). STRING functional enrichment analysis of the 50 proteins showed strong enrichment for biological processes related to lipid metabolism and immune/inflammatory regulation. The most significantly enriched terms included plasma lipoprotein particle remodeling (6 proteins; FDR = 5×10^−6^) and regulation of plasma lipoprotein particle levels (7 proteins; FDR = 5×10^−6^), along with cholesterol homeostasis (6 proteins; FDR = 2.5×10^−4^). In addition, the protein set was enriched for inflammation-related processes, including regulation of inflammatory response (10 proteins; FDR = 1.2×10^−4^) and regulation of macrophage-derived foam cell differentiation (5 proteins; FDR = 8.7×10^−5^), supporting involvement of pathways linking lipid handling, atherosclerotic biology, and systemic inflammatory signaling. Protein–protein interaction analysis further demonstrated that these 50 proteins formed a highly interconnected network. Using coexpression information from the STRING database, a dense subnetwork comprising 31 proteins was identified, with each node exhibiting at least two protein–protein interaction connections, indicating coordinated biological activity within lipid-inflammatory regulatory pathways (Fig 6).

## Discussion

In this study, we developed and validated ProAL50, a proteomics-derived index of AL, and demonstrate that it not only faithfully reproduces the well-established sociodemographic, behavioral, and health correlates of traditional AL, but also outperforms traditional AL in predicting incident chronic disease and mortality across multiple organ systems. Together, these findings position ProAL50 as a biologically grounded, scalable, and clinically translatable alternative to conventional AL measurement.

A central strength of ProAL50 is its strong construct validity. Across sociodemographic characteristics, lifestyle factors, and general and mental health measures, ProAL50 showed patterns of association that closely mirrored those observed for traditional AL. Age, sex, socioeconomic position, neighborhood deprivation, smoking, physical activity, sleep disturbance, fatigue, psychological distress, and inflammatory burden were all consistently related to higher ProAL50 in directions and magnitudes comparable to AL. This concordance supports the interpretation of ProAL50 as a robust marker of cumulative physiological dysregulation rather than a disease-specific or pathway-restricted biomarker. Importantly, these associations were observed despite ProAL50 being derived entirely from circulating proteins, without reliance on traditional clinical risk factors such as blood pressure, lipids, or glucose.

Beyond construct validity, ProAL50 demonstrated superior predictive performance for both disease incidence and mortality. While AL and ProAL50 were similarly associated with overall cancer risk, metabolic disease, chronic lung disease, and chronic kidney disease, ProAL50 consistently yielded larger effect sizes and retained statistical significance in settings where AL associations were attenuated after covariate adjustment. This pattern was particularly evident for ischemic heart disease incidence, several site-specific cancers, and cause-specific mortality outcomes. In mortality analyses, ProAL50 showed markedly stronger associations with all-cause mortality, cancer mortality, cardiometabolic mortality, chronic liver disease mortality, and chronic kidney disease mortality, even in analyses constrained by limited event counts. These findings suggest that ProAL50 captures dimensions of biological vulnerability that extend beyond what is indexed by traditional clinical AL.

The enhanced prognostic performance of ProAL50 likely reflects fundamental differences in what these measures capture biologically. Traditional AL indices are composites of static clinical measurements, many of which reflect downstream consequences of dysregulation and are influenced by short-term physiological states, medication use, and measurement context (2, 3, 5). In contrast, circulating proteomic profiles integrate genetic predisposition, cumulative environmental exposures, behavioral patterns, immune activation, and metabolic regulation, providing a dynamic and systems-level readout of physiological wear and tear (10, 11, 14–17). The strong enrichment of ProAL50 proteins in lipid metabolism and immune–inflammatory pathways underscores this point, highlighting processes central to cardiometabolic disease, atherosclerosis, cancer progression, and organ failure. The highly interconnected protein–protein interaction network further suggests that ProAL50 reflects coordinated biological programs rather than isolated biomarkers.

From a translational perspective, ProAL50 overcomes several key limitations that have constrained the utility of traditional AL. Traditional AL requires multiple heterogeneous measurements, fasting samples, and arbitrary cut-points, and suffers from substantial missingness and poor cross-study comparability (3, 5). In contrast, ProAL50 can be derived from a single plasma sample using standardized proteomic platforms, with consistent scaling and reproducibility across cohorts. The strong external validation observed in the CARDIA cohort further supports its generalizability. These features make ProAL50 particularly well suited for large biobanks, longitudinal cohort studies, and clinical settings where comprehensive clinical laboratory panels are unavailable or impractical.

Several findings also provide insight into the biological embedding of chronic stress. The strong associations of ProAL50 with physical functioning, fatigue, psychological distress, inflammatory markers, and accelerated biological aging suggest that proteomic AL may be especially sensitive to stress-related immune and metabolic dysregulation. The pronounced associations with kidney disease incidence and mortality, as well as liver disease mortality, are consistent with emerging evidence that chronic stress and systemic inflammation play central roles in organ vulnerability and failure (18–20). Conversely, the absence of associations with neurodegenerative disease outcomes may reflect limited statistical power, distinct etiologic pathways, or the need for central nervous system–specific biomarkers (21, 22).

This study has several limitations. First, although ProAL50 was externally validated, further validation in more diverse populations and clinical settings is warranted. Second, while proteomic platforms are increasingly accessible, cost and infrastructure requirements may currently limit widespread implementation, though these barriers are rapidly diminishing. Finally, although we demonstrate strong associations with disease and mortality outcomes, causal inference remains limited; ProAL50 should be interpreted as a marker of vulnerability rather than a direct mechanistic mediator.

In summary, ProAL50 represents a next-generation measure of allostatic load that preserves the conceptual foundation of AL while substantially improving its predictive performance and translational feasibility. By capturing multisystem biological dysregulation through circulating proteins, ProAL50 not only replaces traditional AL in epidemiologic research but also surpasses it as a predictor of chronic disease risk and mortality. These findings support the use of proteomics-based AL as a powerful tool for population health research, risk stratification, and the study of how chronic stress becomes biologically embedded to shape long-term health outcomes.

## Methods

### Study participants

We analyzed data from the UK Biobank, a large population-based cohort of more than 500,000 adults aged 40–69 years enrolled between 2006 and 2010. Participants with available proteomic measurements, allostatic load components, and complete covariate and outcome information were eligible for inclusion. Proteomic profiling was conducted in a random subsample of participants (*n* = 53,013), which has been shown to be broadly representative of the wider UK Biobank cohort (23). Olink proteomic data were provided as Normalized Protein Expression (NPX) values on a log2 scale, after centralized preprocessing and quality control performed by the UK Biobank. Detailed information on sample selection, laboratory processing, and quality control procedures is available in the UK Biobank documentation (https://biobank.ndph.ox.ac.uk/ukb/label.cgi?id=1839).

### AL calculation

We constructed an AL score using eleven physiological biomarkers measured at baseline (**Supplementary Table 1**), following methodologies described previously by Guan and Shen et al (24, 25). The biomarkers encompassed three major physiological domains: cardiovascular (systolic blood pressure, diastolic blood pressure, and pulse rate), inflammatory (C-reactive protein), and metabolic (high-density lipoprotein cholesterol, low-density lipoprotein cholesterol, waist-to-hip ratio, total cholesterol, triglycerides, hemoglobin A1c, and creatinine). Each biomarker was dichotomized using clinically established high-risk thresholds, with participants assigned a value of 1 for high-risk and 0 otherwise. In addition, individuals reporting use of medications for metabolic disorders or hypertension were assigned a high-risk score of 1. The AL score was calculated as the sum of high-risk indicators (range, 0–11), with higher values indicating greater cumulative physiological dysregulation. Participants without sufficient biomarker data to calculate an AL score were excluded from the analysis (N = 12,139). Thus, 40,874 participants were included in the final score construction.

### Model benchmarking

To identify proteomic predictors of AL, we evaluated two regularized regression methods, LASSO and Elastic Net (ENet), using the continuous AL score as the training outcome and standardized protein expression levels as predictors. All models were fitted to the UK Biobank training subset (70%), with preprocessing parameters (median imputation and scaling) estimated exclusively from the training data. Both methods were implemented using the glmnet framework. For LASSO, an L1 penalty was applied to induce sparsity. For Elastic Net, we specified α = 0.7, balancing the sparsity of LASSO with the coefficient stability of ridge regression. Hyperparameter tuning followed a 10-fold cross-validation procedure, and the λ_min_ and λ_1-SE_ criterion were used for feature selection to promote parsimony and generalizability. Proteins with non-zero coefficients at λ_1-SE_ were designated as selected features. Model performance was assessed using cross-validated error, feature selection stability (via 1,000 bootstrap subsamples), and discrimination in the held-out 30% test set. Correlation was used to assess how accurately the training model predicted AL. The Elastic Net method was ultimately selected as the primary modeling approach for downstream analyses based on its balance of predictive accuracy, feature stability, and interpretability.

### Protein feature selection and score construction

Elastic Net regularization was used to identify a parsimonious subset of proteins associated with AL and to construct a standardized proteomic AL score. Protein measurements were preprocessed by the UK Biobank. Missing values were imputed using median imputation, and all predictors were standardized using training-set means and standard deviations. The dataset was randomly split into training (70%) and testing (30%) partitions.

The optimal regularization parameter (λ) was selected via 10-fold cross-validation. As in benchmarking, both λ_min_ and λ_1-SE_ were evaluated, and λ_1-SE_ was chosen to enhance robustness and reduce overfitting. Coefficients were extracted from the final ENet model at λ_1-SE_. Proteins with nonzero coefficients were ranked according to the absolute magnitude of their standardized regression coefficients, reflecting their relative contribution to the model. The top 50 ranked proteins were retained for subsequent analysis. To facilitate comparison across models, coefficients were normalized by dividing each by the sum of their absolute values.

A weighted Elastic-Net proteomic score (ProAL50) was then computed for each participant as:

$${\text{Score}}_{\text{EN}}=\backslash {\text{sum}}_{{\left\{\text{i=1}\right\}}_{\text{i}}^{\{50\}{\text{w}}_{{\text{z}}_{\text{i}}}}}$$

where z_i_ is the standardized level of the i-th protein, and w_i_ is the normalized ENet coefficient for that protein (i.e., w_i_ = β_i_ / Σ|β_j_|). This standardized score reflects the aggregate weighted influence of the top 50 proteins identified by the Elastic Net model. The score was computed in both the training and test cohorts using identical weights derived exclusively from the training set.

### Internal and external validation

Internal validation was conducted to evaluate the robustness of the ProAL50 against alternative constructions of AL. Within the 30% test set of the UK Biobank cohort, two additional AL indices were independently constructed using distinct component selections and scoring schemes. The first index followed a conventional quantile-based approach using established clinical biomarkers, whereas the second index was derived using a reduced set of components aligned with the original AL framework while applying identical scoring rules. Pearson correlation analyses were used to assess the consistency of associations between ProAL50 and each of the two newly constructed AL measures.

External validation was performed in an independent cohort (CARDIA Y25) to assess model generalizability. The ProAL50 score was generated by applying the fixed protein weights and an identical protein list derived from the UK Biobank training set, without model re-estimation. In parallel, AL was reconstructed in the external dataset using the same component definitions and scoring procedures as in the UK Biobank cohort. Associations between ProAL50 and the externally derived AL measure were evaluated using Pearson correlation coefficients.

### Statistical analysis

Associations of sociodemographic and lifestyle factors with AL and the ProAL50 proteomic score were examined using multivariable linear regression. Each outcome (AL or the ProAL50 score) was modeled separately as a continuous dependent variable. Covariates included age group, sex, race/ethnicity, household income, educational attainment, Townsend deprivation index, smoking status, alcohol consumption, physical activity level, and sleep disturbance category. All predictors were coded as categorical variables, with reference categories specified in Table 1. Associations between indicators of general health and mental health with AL and ProAL50, were examined using multivariable linear regression models. Each index was analyzed as a standardized continuous outcome, with regression coefficients representing mean differences relative to the reference category for each exposure. Walking pace, self-rated health, fatigue frequency, ProAgeGap category, neutrophil-to-lymphocyte ratio (NLR), anxiety, depression, and anxiety/depression disorders were modeled as categorical variables based on established definitions. All models were adjusted for the same sociodemographic and lifestyle covariates listed above. Associations between AL, ProAL50 score, and incident disease outcomes, including overall cancer, type 2 diabetes, ischemic heart disease, cerebrovascular disease, emphysema/COPD, chronic liver disease, chronic kidney disease, Alzheimer’s disease, and Parkinson’s disease, were assessed using Cox proportional hazards models. Time-to-event was calculated from baseline to incident diagnosis, death, loss to follow-up, or end of registry linkage. For each exposure–outcome pair, we fitted three hierarchical models: (1) an unadjusted crude model; (2) Model 1, adjusted for demographic, socioeconomic, lifestyle, and behavioral factors; (3) Model 2, which additionally included disease-specific risk factors such as family history, polygenic risk score (PRS), and dietary variables. Hazard ratios (HRs) and 95% confidence intervals (CIs) were reported with corresponding *P* values. Proportional hazards assumptions were evaluated using Schoenfeld residuals, with no significant violations detected. Cox models were also used to examine associations between AL, ProAL50 score, and mortality outcomes, including all-cause mortality and deaths due to overall cancer, type 2 diabetes, Alzheimer’s disease, cerebrovascular disease, ischemic heart disease, emphysema/COPD, chronic liver disease, and chronic kidney disease. For mortality analyses, only crude and Model 1 estimates were presented to avoid overadjustment for potential mediators. The same analytic framework was applied to examine associations with both the incidence and mortality of site-specific cancers, including breast, bowel (colorectal), lung, kidney, prostate, pancreatic, cervical, ovarian, bladder, esophageal, liver, brain, stomach cancers, non-Hodgkin lymphoma, and leukemia, with each cancer type analyzed separately. Functional enrichment analyses were performed to characterize the biological pathways represented by the ProAL50 protein panel. Gene Ontology (GO) biological processes and molecular functions, Kyoto Encyclopedia of Genes and Genomes (KEGG), Reactome, and WikiPathways annotations, as well as protein–protein interaction (PPI) networks, were obtained from the STRING database (version 12.0) using the STRING web interface. Enrichment results were used to support the biological interpretation of the ProAL50 proteomic signature. All analyses were performed using R (version 4.3.0), with statistical significance defined as two-sided *P* < 0.05. *P* values were corrected for multiple comparisons via the FDR using the Benjamini–Hochberg method (26).

## Supplementary Files

This is a list of supplementary files associated with this preprint. Click to download.

- CopyofSupplements02022026.xlsx

## Figures and Tables

**Fig. 1 |:** Development, validation, and application of ProAL50. **a,** Derivation of the ProAL50 proteomic score from 2,922 plasma proteins using penalized regression and bootstrap stability selection in the UK Biobank. **b,** Internal validation in the UK Biobank test set and external validation in the CARDIA Study (Year 25) by comparison with established allostatic load measures. **c,** Application of ProAL50 to sociodemographic, lifestyle, health, disease, cancer, and mortality outcomes using regression and survival analyses.

**Fig. 2 |:** Correlation between ProAL50 and established allostatic load measures. **a,** Correlation between continuous AL and the ProAL50 score in the UK Biobank training set. **b,** Association between quartile-based AL and ProAL50 in the UK Biobank test set. **c,** Correlation between ProAL50 and an AL derived from a reduced biomarker panel. **d,** External validation showing the association between AL and ProAL50 in the CARDIA Study (Year 25). Lines indicate fitted linear regression with 95% confidence intervals; Pearson correlation coefficients (r) and P values are shown.

**Fig. 3 |:** Associations of general and mental health indicators with allostatic load and ProAL50. Multivariable linear regression estimates showing associations of general health and mental health indicators with AL and the ProAL50 proteomic score in the UK Biobank. Regression coefficients represent mean differences relative to the reference category for each indicator. Models were adjusted for demographic, socioeconomic, and lifestyle covariates. Point size denote false discovery rate (FDR)–adjusted significance levels, and open points indicate non-significant associations (FDR > 0.05).

**Fig. 4 |:** Associations of allostatic load and ProAL50 with incident disease risk. Hazard ratios from Cox proportional hazards models showing associations of AL and the ProAL50 proteomic score with incident chronic diseases and overall cancer in the UK Biobank. Models were adjusted for demographic, socioeconomic, lifestyle, and behavioral covariates. Points represent hazard ratios and horizontal bars indicate 95% confidence intervals; numbers of events are shown. Point color encodes exposure (blue = AL, red = ProAL50), and point size encodes FDR-adjusted significance level (<0.05, <0.01, <0.001); open points indicate non-significance (FDR > 0.05).

**Fig. 5 |:** Associations of allostatic load and ProAL50 with mortality outcomes. Hazard ratios from Cox proportional hazards models showing associations of AL and the ProAL50 proteomic score with all-cause mortality and cause-specific mortality in the UK Biobank. Models were adjusted for demographic, socioeconomic, lifestyle, and behavioral covariates. Points represent hazard ratios and horizontal bars indicate 95% confidence intervals; numbers of deaths are shown. Point color encodes exposure (blue = AL, red = ProAL50), and point size encodes FDR-adjusted significance level (<0.05, <0.01, <0.001); open points indicate non-significance (FDR > 0.05).

**Fig. 6 |:** Protein–protein interaction network of the ProAL50 proteomic signature. Protein–protein interaction (PPI) network of a highly interconnected subset of proteins in the ProAL50 model, restricted to nodes with at least two connections, constructed using experimentally supported interactions from the STRING database. Nodes represent proteins, and edges represent known or predicted functional associations, with color intensity indicating the number of connections.

**Table 1.** Sociodemographic and lifestyle determinants of AL and ProAL50 (N=37,089)

		AL		ProAL50	
	N (%)	Coefficient (95%CI)	P value	Coefficient (95%CI)	P value
Age					
<=57	16,919 (45.62)	Ref		Ref	
>57	20,170 (54.38)	0.65 (0.61, 0.68)	<0.001	0.12 (0.11, 0.13)	<0.001
Gender					
Female	19,468 (52.49)	Ref		Ref	
Male	17,621 (47.51)	0.98 (0.95,1.01)	<0.001	0.25 (0.24, 0.26)	<0.001
Race					
White	34,671 (93.48)	Ref		Ref	
Black	758 (2.04)	0.09 (−0.03, 0.20)	0.15	−0.05 (−0.07, −0.03)	<0.001
Asian	850 (2.29)	0.23 (0.12, 0.34)	<0.001	0.06 (0.04, 0.07)	<0.001
Mix or others	688 (1.85)	−0.08 (−0.20, 0.04)	0.21	−0.02 (−0.04, −0.01)	0.032
Income					
<=30999	15,473 (41.72)	Ref		Ref	
>30999	16,116 (43.45)	−0.19 (−0.23, −0.15)	<0.001	−0.03 (−0.04, −0.02)	<0.001
Education					
High school or less	16,416 (44.26)	Ref		Ref	
College or professional	13,940 (37.59)	−0.22 (−0.26, −0.18)	<0.001	−0.03 (−0.04, −0.02)	<0.001
Townsend					
Low	18,213 (49.11)	Ref		Ref	
High	18,826 (50.76)	0.09 (0.05, 0.12)	<0.001	0.02 (0.01, 0.03)	<0.001
Smoke					
Never	20,158 (54.35)	Ref		Ref	
Previous	12,855 (34.66)	0.17 (0.14, 0.21)	<0.001	0.03 (0.02, 0.04)	<0.001
Current	3,959 (10.67)	0.14 (0.09, 0.20)	<0.001	0.06 (0.05, 0.07)	<0.001
Alcohol					
Never	7,461 (20.12)	Ref		Ref	
Moderate	13,568 (36.58)	−0.21 (−0.25, −0.16)	<0.001	−0.05 (−0.06, −0.04)	<0.001
Heavy	16,026 (43.21)	−0.27 (−0.31, −0.22)	<0.001	−0.07 (−0.09, −0.06)	<0.001
Physical activity					
Low	5,851 (15.78)	Ref		Ref	
Moderate	12,032 (32.44)	−0.29 (−0.34, −0.24)	<0.001	−0.06 (−0.07, −0.05)	<0.001
High	12,115 (32.66)	−0.48 (−0.53, −0.43)	<0.001	−0.10 (−0.11, −0.09)	<0.001
Sleepless					
Never	8,964 (24.17)	Ref		Ref	
Sometimes	17,624 (47.52)	0.10 (0.06, 0.14)	<0.001	0.02 (0.01, 0.03)	<0.001
Usually	10,467 (28.22)	0.22 (0.17, 0.26)	<0.001	0.04 (0.03, 0.05)	<0.001

## Data Availability

Data underlying this study were accessed through the UK Biobank under application number 94449. Researchers who meet UK Biobank eligibility criteria may obtain access to the same data by submitting an independent application via the UK Biobank Access Management System (https://www.ukbiobank.ac.uk/enable-your-research/apply-for-access).

## Code availability

Analysis scripts used for participant selection, data preparation, and statistical modelling may be shared by Prof. Hua Zhao (qqx7mw@virginia.edu) upon reasonable request. Release of code is contingent on approval from the relevant institutional and ethics committees, and approved requests will be processed within six months.
